# Effects of High Temperature Stress on the Physiological and Biochemical Characteristics of *Paeonia ostii*

**DOI:** 10.3390/ijms241311180

**Published:** 2023-07-06

**Authors:** Erman Hong, Xuanze Xia, Wen Ji, Tianyao Li, Xianyi Xu, Jingran Chen, Xia Chen, Xiangtao Zhu

**Affiliations:** College of Jiyang, Zhejiang AF University, Zhuji 311800, China; mandaa98@163.com (E.H.); xiaxuanze928@163.com (X.X.); jiwenn@163.com (W.J.); 13065665778@163.com (T.L.); xuxiannyiii@163.com (X.X.); m15325506050@163.com (J.C.)

**Keywords:** peony, high temperature stress, antioxidant enzymes, photosynthesis, heat resistance, PLS-DA

## Abstract

In order to explore the effects of high temperature stress on the physiological characteristics of *Paeonia ostii*, the *Paeonia ostii* were subjected to 25 °C, 35 °C, 38 °C, and 40 °C for 7 days. Meanwhile, the physiological indicators of oxidative stress (hydrogen peroxide, H_2_O_2_; malondialdehyde, MDA; relative electrical conductivity, REC), antioxidant enzyme activity (superoxide dismutase, SOD; ascorbate peroxidase, APX; catalase, CAT; peroxidase, POD), photosynthetic pigment content (chlorophyll a, Chla; chlorophyll b, Chlb), photosynthetic characteristics (net photosynthetic rate, Pn; intercellular CO_2_ concentration, Ci; stomatal conductance, Gs; transpiration rate, Tr), and osmoregulatory substances content (soluble protein, SP; soluble sugar, SS) were determined. The results showed that, with the increase in temperature and stress time, the H_2_O_2_ content, MDA content, REC value, CAT activity, and APX activity increased, while Chla content, Chlb content, SS content, and SP content decreased. With the extension of stress time, the SOD activity, POD activity, and Tr value of each high temperature stress group first increased and then decreased; Ci first decreased, then increased, and then decreased; meanwhile, Pn and Gs showed an overall downward trend. PLS-DA (partial least squares discriminant analysis) was used to analyze the changes in physiological and biochemical indexes of peony leaves under 40 °C stress for different days. SOD was found to be the biggest factor affecting the changes in physiological and biochemical indexes of peony leaves treated with different days of stress.

## 1. Introduction

The peony (*Paeonia suffruticosa* Andr.) is a perennial deciduous shrub with beautiful and dignified flowers, known as the “national color and fragrance” and “king of flowers”. Its root has anti-hypertensive and anti-inflammatory properties [1]. The climate in the south of the Yangtze River is humid and hot, and the temperature in summer is high. The prolonged high temperature causes the peony leaves to curl and wilt, which is unfavorable for growth and affects large-scale production. *P. ostii* is one of the main varieties of the Jiangnan peony, which is more resistant to moisture and heat [2,3] and can adapt to the high temperature and high humidity environment in the Jiangnan area. Therefore, it is of great significance to explore its heat resistance mechanism under high temperature stress.

In the context of global warming and the increasing frequency of extreme temperatures, high temperature is considered to be an important environmental factor affecting plant growth. Heat stress in plants refers to the phenomenon whereby the ambient temperature rises above a critical threshold for a period of time, causing irreparable damage to plant growth and development [4]. Heat stress significantly affects plant developmental processes, such as seed germination, vegetative growth, and reproductive production [5]. In addition, heat stress can affect important plants’ physiological processes, namely photosynthesis and respiration rates, stomatal conductance, and leaf water potential homeostasis [6]. It is widely accepted that an increase in LMA is a common response to environmental stress [7]. Traditionally, high LMA has also been interpreted as a trait that increases a leaf’s structural resistance, as it protects leaves from phytosexual or mechanical damage [8]. Under normal circumstances, plants will produce ROS during normal metabolism, and there is a dynamic balance between ROS production and antioxidants [9]. However, when plants are under stress, ROS production is accelerated, and the balance is disturbed. Excessive ROS can lead to membrane lipid peroxidation, protein oxidation, enzyme inactivation, and DNA and RNA damage, resulting in plant injury. When high temperature causes a large accumulation of ROS in plants, the antioxidant defense system is activated to remove excessive ROS, thereby protecting plants from oxidative stress [10]. At the same time, osmoregulatory substances such as SS, SP, proline (Pro), etc., can work together with antioxidant enzymes to alleviate plant damage caused by high temperature stress. Some studies have shown that peonies reduce oxidative damage and osmotic stress by increasing the activity of antioxidant enzymes and accumulating Pro, thereby improving the plant’s heat tolerance [11].

At present, the research on peonies mainly focuses on pharmacological effects and germplasm resources, and there are relatively few studies on its physiological mechanism under high temperature and the breeding of high temperature-resistant cultivars [3]. Previous studies have found that light energy distribution in peony leaves is affected by high temperature, which can cause photoinhibition of PSⅡ, induce irreversible inactivation of the PSⅡ reaction center [12], and hinder electron transfer [13]. However, peony cultivars resistant to high temperature stress have a relatively low degree of time inhibition under high temperature stress and can maintain relatively high PSⅡ actual light energy conversion efficiency [14]. Ji et al. [15,16] investigated the changes in photosynthetic characteristics of *P. ostii* under high temperature stress and found that high temperature damaged the photosynthetic capacity and photosynthetic mechanism of peony leaves. At the same time, some studies have shown that exogenous additives can alleviate the photosynthetic characteristics of peonies under high temperature stress [17,18,19,20,21]. In terms of physiology and biochemistry, Qian et al. [22] treated peonies at different temperatures and found that the degree of damage of peonies was related to the intensity of high temperature stress. Li et al. [23] used principal component and membership function analysis to establish a comprehensive identification and evaluation system of peony heat tolerance, which is suitable for the screening and evaluating of peony cultivar resources with heat tolerance. Wang et al. [11] discussed the physiological response mechanism of peonies under high temperature. In recent years, researchers have started to study the mechanism of high temperature resistance of peonies from the molecular point of view [24,25,26,27].

According to previous studies, in response to high temperature stress, antioxidant enzymes, photosynthetic pigments, photosynthetic properties and osmoregulatory substances all change to mitigate the damage caused by high temperature. However, there are still few related reports in Peony, and the physiological and biochemical changes in peony treated for different days under different high temperature stress are still unclear. Therefore, in this study, the activities and contents of antioxidant enzymes (SOD, POD, CAT, APX), photosynthetic pigments (Chl a and Chl b), photosynthetic properties (Ci, Pn, Gs, Tr), and osmoregulatory substances (sp and ss) in peonies subjected to high temperature for different days were investigated and also analyzed using PLS-DA; meanwhile, scanning electron microscopy was used to observe the structure of peony leaves after high temperature treatment to study the effects of high temperature on the physiological and biochemical characteristics of peony leaves and to provide theoretical basis for the screening of peony germplasm resources and the cultivation of new varieties.

## 2. Results

### 2.1. Effects of High Temperature Stress on ROS Accumulation and Lipid Peroxidation in Peony Leaves

**Figure 1:**
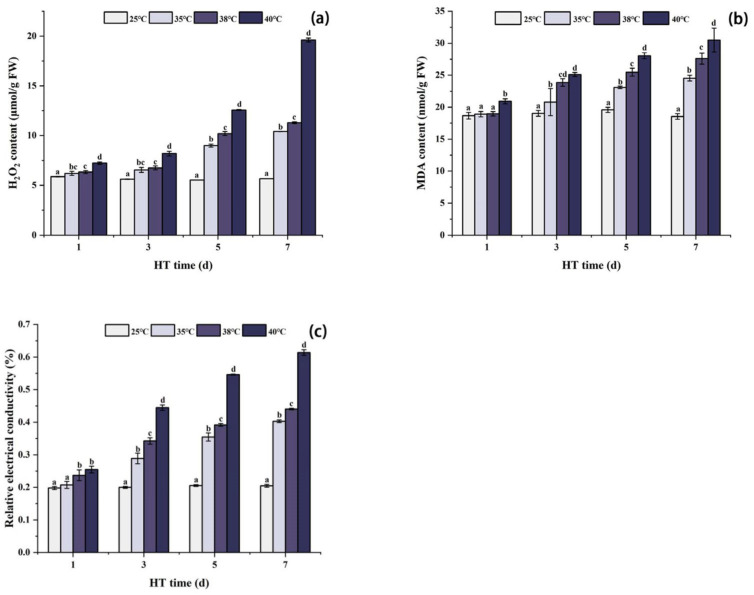
Changes in H_2_O_2_ content, MDA content, and REC value in peony leaves under high temperature stress (**a**–**c**). FW, fresh weight; different letters in the same column indicate significant differences at the 0.05 level.

As shown in Figure 1a, the content of H_2_O_2_ increased with the increase in stress temperature and treatment time. Beginning from the first day of high temperature stress, the H_2_O_2_ content in peony leaves under each high temperature treatment was significantly higher than that of CK (25 °C). After 7 days of stress, the content of H_2_O_2_ at 35 °C, 38 °C, and 40 °C was significantly higher than that in the CK group, increasing by 83.7%, 99.2%, and 246.1%, respectively.

High temperature stress increased the MDA content and REC value, and the higher the temperature and the longer the stress time, the higher the MDA content and REC value. After 1 day of high temperature stress, the MDA content and REC value changed slightly under each high temperature treatment. The MDA content under 35 °C and 38 °C stress was not obviously different from CK, while the MDA content under 40 °C stress was significantly higher. The REC value under 35 °C stress was not significantly different from CK, but the REC value under 38 °C and 40 °C stress was significantly higher. After 3 and 5 days of stress, the MDA content and REC value of each high temperature treatment group were significantly higher than those of the CK group. After 7 days of stress, the MDA content of each high temperature treatment group reached the maximum, which was significantly higher than that in the CK group, increasing by 32.4%, 48.8%, and 64.5%, respectively. At the same time, the REC value of each group also reached the maximum, which was significantly higher than that in the CK group, increasing by 96.8%, 115.1%, and 199.9%, respectively (Figure 1b,c).

### 2.2. Effects of High Temperature Stress on the Activities of Antioxidant Enzymes in Peony Leaves

**Figure 2:**
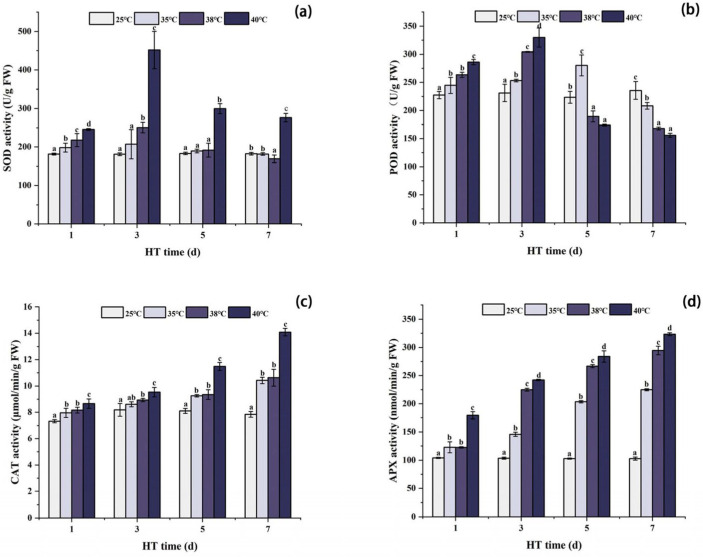
Changes in antioxidant enzyme activities in peony leaves under high temperature stress. (**a**) Superoxide dismutase (SOD); (**b**) ascorbic acid peroxidase (APX); (**c**) catalase (CAT); (**d**) peroxidase (POD). FW, fresh weight; different letters in the same column indicate significant differences at the 0.05 level.

Figure 2 shows that SOD, POD, CAT, and APX of each high temperature treatment group were significantly higher than those of CK at 1d of high temperature stress. The SOD activity of the 35 °C, 38 °C, and 40 °C treatment groups reached the maximum after 3 days of stress, and the POD activity of the 38 °C and 40 °C treatment groups also reached the maximum, while the POD activity of the 35 °C treatment group reached the maximum after 5 days of stress. After 7 days of stress, the CAT and APX activities of each high temperature stress group reached the maximum, and the SOD and POD activities reached the minimum. The POD activity of each high temperature stress group was significantly lower than that in the CK group. Although the SOD activity under 40 °C stress was still significantly higher than that in the CK group, the SOD activity under 35 °C and 38 °C stress was lower than that in the CK group.

### 2.3. Effects of High Temperature Stress on the Photosynthetic Pigment Content of Peony Leaves

**Figure 3:**
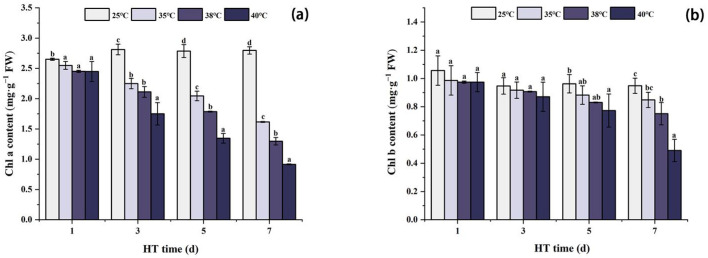
Changes in chlorophyll content of peony leaves under high temperature stress. (**a**) Chla content; (**b**) Chlb content. FW, fresh weight; different letters in the same column indicate significant differences at the 0.05 level.

Figure 3 shows that under the same high temperature stress, the Chla content decreases with the stress time. Compared with CK in the same period, Chla content was the lowest at 7 days of stress, and decreased by 42.2%, 53.6%, and 67.3% at 35 °C, 38 °C, and 40 °C, respectively. The trend of Chlb content was similar to that of Chla; under the same high temperature stress, Chlb content showed a decreasing trend with the extension of stress days. Compared with CK in the same period, Chlb content at 40 °C decreased significantly by 19.7% on the 5th day of stress. On the 7th day of stress, Chlb content at 38 °C and 40 °C decreased by 20.8% and 48.3%, respectively, compared with CK.

### 2.4. Effects of High Temperature Stress on the Photosynthetic Characteristics of Peony Leaves

**Figure 4:**
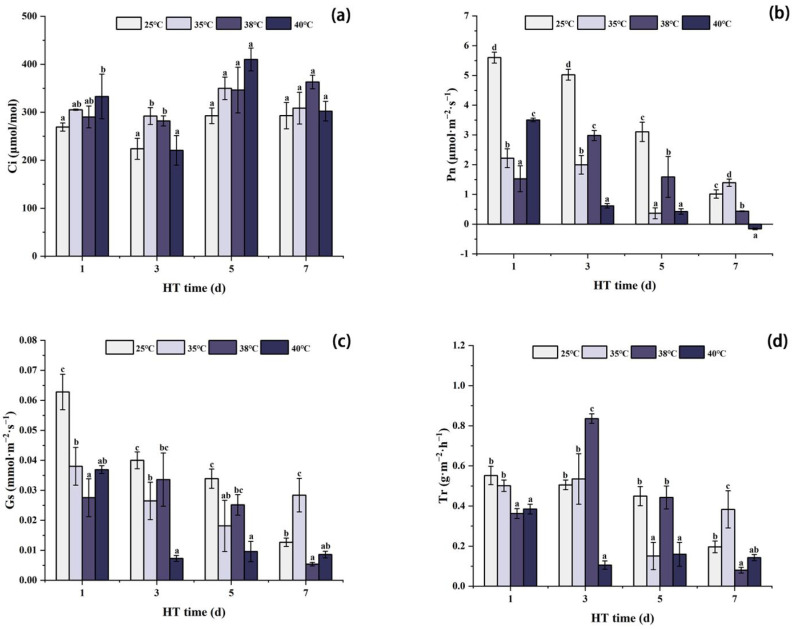
Changes in photosynthetic characteristics of peony leaves under high temperature stress. (**a**) Intercellular CO_2_ content (Ci); (**b**) net photosynthetic rate (Pn); (**c**) stomatal conductance (Gs); (**d**) transpiration rate (Tr). Different letters in the same column indicate significant differences at the 0.05 level.

As shown in Figure 4, the photosynthetic characteristics of peony leaves in each temperature treatment group changed with the high temperature stress treatment. With the extension of high temperature treatment time, Ci first decreased, then increased, and then decreased. After 1 day of stress, the Ci of each high temperature treatment group was higher than that in the CK group, and the Ci of 40 °C was significantly higher than that of CK. After 3 days of stress, the Ci of each high temperature treatment group decreased. Although the Ci at 40 °C was slightly lower than that of CK, the Ci at 35 °C and 38 °C was still significantly higher than that of CK. After 5 days of stress, Ci of each temperature treatment group increased, which was higher than that of CK. After 7 days of stress, the Ci of each high temperature treatment group decreased.

The changes in Pn and Gs were similar and showed an overall decreasing trend with the increase in high temperature treatment time. After 1 day of stress, Pn and Gs of each high temperature treatment group were significantly lower than those of CK, and the values of Pn and Gs at 38 °C were the lowest. After 3 days of stress, Pn and Gs continued to decrease at 35 °C and 40 °C, while they were increased at 38 °C compared with 1 day, but still significantly lower than those of CK. After 5 days of stress, Pn and Gs of each high temperature treatment group decreased, which were significantly lower than those of CK. After 7 days of stress, Pn and Gs at 38 °C and 40 °C continued to decrease, while Pn and Gs at 35 °C were higher than those at 5 days, and significantly higher than those of CK.

As shown in Figure 4d, the results of Tr showed an overall trend of first increasing and then decreasing with the extension of high temperature treatment time. After 1 day of stress, Tr of each high temperature treatment group was significantly lower than that of CK. After 3 days of stress, compared with 1d, Tr at 40 °C decreased, which was significantly lower than that of CK at the same period. Under the stress conditions of 35 °C and 38 °C, Tr was increased, and Tr at 38 °C was significantly higher than that of CK. After 5 days of stress, Tr at 40 °C increased slightly compared with that at 3 days, while Tr at 35 °C and 38 °C decreased, and Tr at 35 °C was significantly lower than that of CK. After 7 days of stress, compared with 5 days of stress, the Tr of 35 °C stress increased, which was significantly higher than that of CK, while the Tr of 38 °C and 40 °C stress decreased, and the Tr of 38 °C stress was significantly lower than that of CK.

### 2.5. Effects of High Temperature Stress on Changes in Osmotic Regulators in Peony Leaves

**Figure 5:**
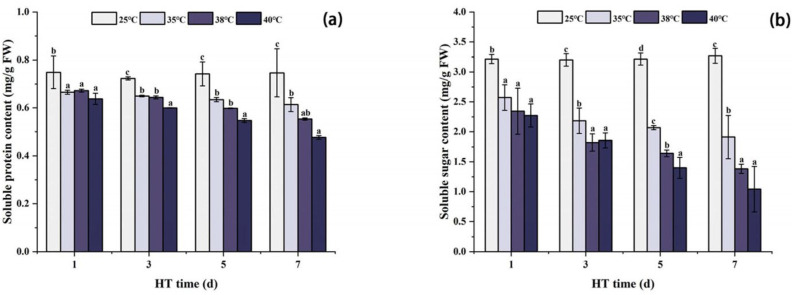
Changes in osmotic regulator content in peony leaves under high temperature stress. (**a**) Soluble protein content. (**b**) Soluble sugar content. FW, fresh weight; different letters in the same column indicate significant differences at the 0.05 level.

As shown in Figure 5, the changes in SP and SS contents in peony leaves under high temperature stress were similar, and both showed a decreasing trend with the extension of high temperature stress time. Compared with CK, the contents of SP and SS were the lowest in each high temperature treatment group after 7 days of stress. Compared with CK, SP at 35 °C, 38 °C, and 40 °C decreased by 17.7%, 25.9%, and 36.2%, respectively, and SS at 35 °C, 38 °C, and 40 °C decreased by 41.5%, 57.7%, and 68.1%, respectively.

### 2.6. Correlation Analysis of Factors under Different Stress Temperatures

Correlation analysis of the indicators of *P. ostii* at 40 °C was performed, and the results are shown in Table 1.

At 40 °C, there was a very significant positive correlation between any two of the five indicators of H_2_O_2_, MDA, REC, CAT, and APX; between any two of the four indicators of ss, sp, Gs and Tr; between Chla, Chlb, SS, and sp; and between any two of the three indicators of Gs, Tr and Pn. Meanwhile, Chla was very positively correlated with Gs and Tr, and Pn was very positively correlated with Chla, SS, and sp. Then, Chlb was positively correlated with Pn, Gs, and Tr.

H_2_O_2_ was very significantly negatively correlated with Chla, Chlb, ss, and sp. In addition, REC, MDA, CAT, and APX were very significantly negatively correlated with Pn, Chla, Chlb, ss, and sp, and CAT, APX, REC, and SOD were very significantly negatively correlated with Gs and Tr. There was a significant negative correlation between H_2_O_2_ with POD, Pn, and Gs; MDA with Gs and Tr; and POD with Ci.

The indicators in Table 1 represent different meanings, and the complex correlation between them indicates that the change in heat tolerance of peonies cannot be evaluated by a single indicator, and multiple indicators should be integrated for analysis.

### 2.7. PLS-DA Analysis of Different Days of High Temperature Stress at 40 °C

**Figure 6:**
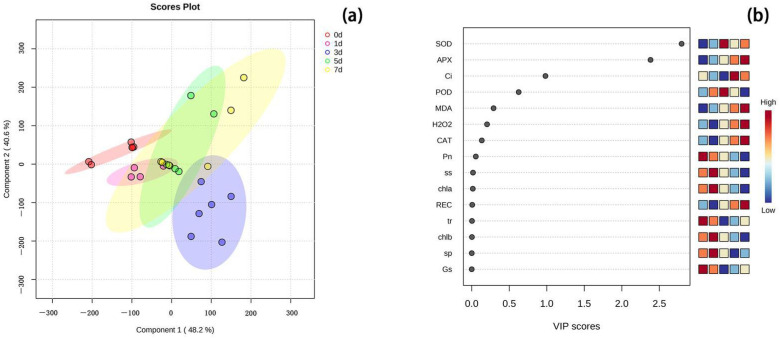
PLS-DA analysis and related VIP scores of various indices of peony leaves under high temperature stress at 40 °C for different stress days. (**a**) Score plot of PLS-DA analysis based on each value for different days of 40 °C stress. The different color spaces represent a separate group. (**b**) The score map of each physiological and biochemical index identified by VIP score; the physiological and biochemical index with a score greater than 1 has reference value. The indicator with the highest VIP score was the variable with the largest differential contribution in the model.

This experiment analyzed the PLS-DA of various physiological and biochemical indexes of peonies leaves under high temperature stress of 40 °C, which is a discriminant analysis method in multivariate data analysis technology and is widely used in genomics, proteomics, and metabolomics [28]. As shown in the PLS-DA model (Figure 6), various indices of peony leaves under high temperature stress at 40 °C were different in different stress days. The VIP score map showed that SOD was the most important contribution model, followed by APX and Ci.

### 2.8. Leaf Anatomy during Different Days of High Temperature Stress at 40 °C

**Figure 7:**
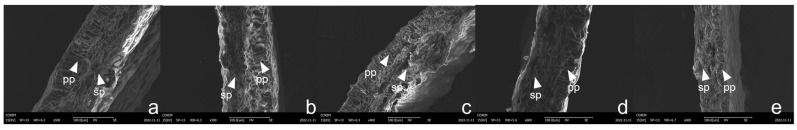
Scanning structure of leaves treated at 40 °C for different days. (**a**) Treatment at 40 °C for 0d; (**b**) treatment at 40 °C for 1d; (**c**) treatment at 40 °C for 3d; (**d**) treatment at 40 °C for 5d; (**e**) treatment at 40 °C for 7d; pp: palisade parenchyma; sp: spongy parenchyma.

As shown in Figure 7, the SEM observation shows that the structure of leaf midvein will extend, along with the time of high temperature stress, to be long and loose. The longitudinal section of the leaf surface showed that the mesophyll tissue of *P. Ostii* was composed of fence tissue and sponge tissue. With the extension of high temperature stress time, the arrangement of fence tissue changed, and the structure of sponge tissue was loose. As shown in Table 2, leaf LMA and LD at a high temperature of 40 °C had similar trends with MT/LT, rising first and then falling back to healthy levels. PT/LT decreased, and ST/LT increased on day 0 and day 5 of the heat stress, and PT/LT increased on day 1 and day 5 of the heat stress. From day 5 to day 7 of the heat stress, PT/LT significantly decreased, and ST/LT significantly increased.

## 3. Discussion

### 3.1. Changes in ROS Accumulation and Lipid Peroxidation in Peony Leaves under High Temperature

High temperature stress will affect the PSII of plants, resulting in a decrease in photochemical efficiency, the inhibition of electron transport, and excess of light energy, thus forming a large number of reactive oxygen species, which will change the permeability of cell membrane and cause membrane lipid peroxidation, causing damage to plants [29,30]. In this experiment, the H_2_O_2_ content increased with the increase in high temperature stress temperature and treatment time, which was consistent with the results of previous studies on tomatoes (*Solanum lycopersicum* L) [31] and sorghum *(Sorghum bicolor* L. *Moench)* [32]. Membrane lipid peroxidation can cause the accumulation of MDA, the final product of lipid peroxidation in plants, so the level of MDA can reflect the degree of cell membrane damage [33]. At the same time, the structure of the cell membrane is destroyed, which significantly increases the membrane’s permeability, resulting in a large amount of extravasation of intracellular electrolyte solution and some small molecular ions, which results in an increase in the conductivity of the tissue leachate [34]. Therefore, REC can be used to determine the extravasation of electrolytes in plant cells and the degree of cell membrane damage [35]. The results of this experiment showed that H_2_O_2_, MDA, and REC were significantly positively correlated under high temperature stress, and the levels of MDA and REC increased with the stress, confirming that high temperature stress damaged the membrane structure of peony cells. These results were consistent with the research results of Wang et al. [11].

### 3.2. Changes in Antioxidant Enzyme Activities in Peony Leaves under High Temperature

If there is an imbalance between ROS production and scavenging in plants, plant cells will produce oxidative stress, which will eventually lead to cell death, and then inhibit plant growth and development [36]. ROS accumulation in plants is controlled by a complex antioxidant defense system [29]. SOD, POD, APX, and CAT are the key enzymes that constitute the antioxidant defense system. In the enzymatic system, SOD, as the first line of defense of a plant’s enzymatic antioxidant system, can catalyze the dismutation reaction of 2 molecules’ O_2_^−^ to generate H_2_O_2_ and O2, and H_2_O_2_ is then catalyzed by CAT, POD, APX, and other antioxidant enzymes to generate H_2_O to achieve the purpose of scavenging ROS [37,38]. Therefore, in this experiment, the increase rates of MDA and REC in each temperature group were higher than those of H_2_O_2_, indicating that H_2_O_2_ was partially removed by antioxidant enzymes. Previous studies have shown that the antioxidant defense system is initiated by various external environmental pressures [9]; during abiotic stress, especially environmental stress (e.g., UV radiation), a plant produces ROS when the plant is exposed to stress, and plant-produced antioxidants, flavonoids, and secondary metabolites play the role of protecting the plant for detoxifying ROS and protecting the plant from abnormal conditions (i.e., stress) and to aid in protein and amino acid stabilization [39,40,41]. Consistent with this conclusion, the activities of antioxidant enzymes were increased under heat stress for a certain period of time (3d) in this experiment. This indicates that peonies can reduce the damage of reactive oxygen species by enhancing the activities of antioxidant enzymes under 3d heat stress, but the activities of APX and CAT still show an increasing trend, while the activities of SOD and POD decreased under long-term (5d–7d) high temperature stress, which is different from the results of previous studies [17,42], indicating that different antioxidant enzymes were sensitive to different temperatures and activated at different temperature ranges, and that activation occurs at different temperature ranges [43]. At the same time, under high temperature stress conditions, H_2_O_2_, MDA, REC, CAT, and APX are positively correlated with each other, indicating that a large amount of H_2_O_2_ induces a significant increase in the activity of antioxidant enzymes involved in ROS scavenging [44,45].

### 3.3. Changes in the Photosynthetic Capacity of Peony Leaves under High Temperature

Chloroplasts are extremely sensitive to high temperature stress in photosynthesis [46,47]. Studies have shown that high temperature can inhibit photosynthetic pigment synthesis in peony seedling leaves [17]. The chlorophyll content measured in this experiment showed that the chlorophyll content decreased with increasing stress, which was consistent with the results of previous studies [11]. Meanwhile, correlation analysis results showed that Chla and Chlb of peony leaves subjected to high temperature stress were very significantly negatively correlated with H_2_O_2_, MDA, and REC, indicating that chlorophyll may be affected by H_2_O_2_ under high temperature stress, resulting in reduced content [48]. At the same time, studies have shown that high temperature stress promotes the activity of chlorophyll-degrading enzymes, thereby accelerating chlorophyll degradation [49].

Pn can directly represent the photosynthetic capacity of an individual leaf [50]. Reduced photosynthetic capacity is associated with lower chlorophyll content [51], which allows leaves to capture less light and thus reduces Pn. In addition, stomatal limitation and non-stomatal limitation are two of the important factors leading to the decrease in the photosynthetic capacity of plants [52]. The gas exchange parameters of *P. ostii* in this experiment showed that the Pn index of *P. ostii* in the first 3 days was limited by stomatal factors. Under high temperature conditions, the decrease in Gs led to the decrease in Tr and Ci, indicating a decrease in plant stomatal conductance, the closure of stomata, a reduction in plant water dissipation, a reduction in the mesophyll cells’ absorption of carbon dioxide, and a further reduction in Pn. At 3d–7d, Pn and Gs decreased significantly, and Ci increased significantly under the three high temperature stresses, indicating that the decrease in Pn in *P. ostii* was not caused by insufficient CO_2_, but by non-stomatal factors. These results were consistent with the results of maize [53] and paeoniflora [54]. Studies have shown that Rubisco activase plays a key role in photosynthesis under heat stress conditions (non-stomatal limitation) [55]. High temperature inactivates the electron acceptor and donor sides of PSII, inactivates enzymes in the Calvin cycle, reduces Rubisco activity, and leads to heat inactivation of Rubisco [56,57]. Rubisco activase can be reversibly inhibited when exposed to moderate high temperature stress, but when exposed to prolonged high temperature, Rubisco activase activity can be irreversibly inhibited due to the insolubility of the Rubisco activase protein and its own degradation [55], thereby affecting photosynthesis. Therefore, the decrease in Pn at 3D–7D may be related to Rubisco activase activity.

### 3.4. Changes in Osmotic Regulatory Substances in Peony Leaves under High Temperature

In addition to antioxidants, osmoregulatory substances (OA) can resist the damage caused by external stress to plants by regulating cell osmotic balance [58,59]. As an OA, SS content can reduce the thermal sensitivity of photosynthetic electron transport [60], protect photosynthetic organs such as chloroplasts from high temperature stress [61], maintain cell osmotic potential, and reduce cytoplasmic membrane damage [61]. Studies have shown that high temperature stress can change sugar metabolism in peonies and promote the accumulation of soluble sugar [22,42]. In this experiment, the content of SS showed a decreasing trend with the increase in stress time, which was different from the previous conclusion [22,42]. High concentrations of H_2_O_2_ generated by high temperatures oxidize proteins in the Calvin cycle, such as cysteine (-SH) or methionine (-SCH3) residues, further inactivating enzymes in the Calvin cycle [62]. Calvin cycle is a carbon-fixation pathway in photosynthesis that provides essential monosaccharides for sucrose synthesis [63]. Therefore, H_2_O_2_ can affect the content of SS and other carbohydrates in plants by affecting the Calvin cycle. Under high temperature stress, SS is negatively correlated with H_2_O_2_, and positively correlated with Pn, Chla, and Chlb, which also confirms the effect of H_2_O_2_ and photosynthesis on SS. Therefore, SS content in this experiment was reduced under high temperature stress.

As an osmoregulatory substance, soluble proteins mostly exist in the form of enzymes in plants and can participate in many physiological activities of plants, such as improving cell osmotic potential and preventing cytoplasmic dehydration. Soluble proteins can be used as a basis for evaluating a plant’s metabolic capacity. Studies have shown that peonies can increase soluble protein content in leaves under short-term high temperature stress, thereby reducing water loss of cells and maintaining cell morphology. However, with the increase in stress time, the regulation of this pathway is impaired, and soluble protein content decreases [42]. Consistent with this conclusion, in the present experiment, the soluble protein content showed a decreasing trend with the stress time.

## 4. Materials and Methods

### 4.1. Materials and Treatment

In this study, strong and consistent 4-year old peonies (*P. ostii)* were planted in a plastic basin with an upper diameter of 28 cm, a lower diameter of 19 cm, and a height of 23 cm. The substrate was composed of garden soil, sand, and perlite (mass ratio: 5:3:2), and water and fertilizer management was normal. Four-year-old peonies with basically the same growth and size were selected. The control group was treated at 25 °C, and the experimental group was treated at 35 °C, 38 °C, and 40 °C, repeated three times. During the experiment, the air humidity in the incubator was set at 70%, the light intensity was set at 8000 lx, and the light and night were 14 h/10 h each day. The vapor pressure deficit was constant at all temperatures. Samples (the first pair of leaves under the top bud) were collected at 0, 1, 3, 5, and 7d after treatment, and three plants were randomly selected from each treatment as replicates. During sampling, photosynthetic indices were measured first, and then leaves were taken for chlorophyll content determination and antioxidant enzyme activity determination. Each measurement was replicated three times.

### 4.2. Determination of H_2_O_2_ Content, MDA Content, and REC Value

H_2_O_2_ content and MDA content were determined using a kit (Suzhou Keming Biotechnology Co., Ltd., Suzhou, China), and the procedure was repeated three times. The specific steps were as follows:

H_2_O_2_ content: 0.1 g (W) of the leaves of different treatment groups were added with reagents, and the supernatant was extracted for the test. At the same time, the same volume of reagents was used as A control, and the microplate reader was used for determination: H_2_O_2_ content (μmol/g fresh weight) = 2.67 × (ΔA − 0.0006) ÷ W, (ΔA = A determination − A control).

MDA content: 0.1 g (W) leaves of different treatment groups were extracted, and the absorbance values at 532 nm and 600 nm were measured and calculated: MDA content (nmol/g fresh weight) = 51.6 × ΔA ÷ W, (ΔA = A532 − A600).

REC was determined using the conductivity meter method. First, 0.1 g leaves were ground into fine powder in liquid nitrogen, and the zero value of conductivity (E0) was measured after adding distilled water. The initial value of conductivity (E1) was measured after the sample was static in the dark for 3h, and the final value of conductivity (E2) was measured after 10 min of boiling water bath. REC% = (E1 − E0)/(E2 − E0) ∗ 100%. The procedure was repeated three times.

### 4.3. Determination of Antioxidant Enzyme Activity

SOD activity, POD activity, CAT activity, and APX activity were determined using kits (Suzhou Keming Biotechnology Co., Ltd., Suzhou, China), and the procedure was repeated three times. The specific steps were as follows:

SOD activity: 0.1 g (W) of leaf supernatant from different treatment groups was selected as the experimental group, and the same volume of distilled water was selected as the control group. The absorbance values at 560 nm of the two groups were measured and calculated: percentage of inhibition = (A control tube−A assay tube) ÷ A control tube 100%; SOD (U/g fresh weight) = 11.11 × percentage inhibition ÷ (1 − percentage inhibition) ÷ W.

POD activity: The supernatant of 0.1 g (W) leaves from different treatment groups was extracted and measured at a wavelength of 470 nm A1 (absorbance at 1 min) and A2 (absorbance at 2 min), and POD activity was calculated: POD (U/g fresh weight) = 4000 × ΔA ÷ W, ΔA = A2 − A1.

CAT activity: 0.1 g (W) of the leaves of different treatment groups were added with reagents, and the supernatant was extracted for the test. At the same time, the same volume of reagents was used as A control, and the microplate reader was used for determination and calculation: CAT (μmol/min/g fresh weight) = 8.9 × (ΔA − 0.0013) ÷ W, ΔA = A control − A determination.

APX activity: The supernatant of 0.1 g (W) leaves from different treatment groups was extracted and measured at a wavelength of 290 nm A1 (absorbance at 10 s) and A2 (absorbance at 130 s), and APX activity was calculated: APX (nmol/min/g fresh weight) = 1786 × ΔA ÷ W, ΔA = A1 − A2.

### 4.4. Determination of Photosynthetic Pigment Content

The chlorophyll was extracted using the absolute ethanol extraction method, and absolute ethanol was used as blank control. The absorbance of chlorophyll extract in the experimental group and absolute ethanol in the control group at the wavelength of 663 nm and 645 nm was measured, respectively. Each replicate was measured three times at different wavelengths, and the average value was taken.

The chlorophyll content was calculated using the following formula:

Chla content = (12.7D663 nm − 2.69D645 nm) × V/(1000 × m);

Chlb content = (22.9D645 nm − 4.68D663 nm) × V/(1000 × m).

where D663 nm and D645 nm are the absorbance of the solution to be measured at 663 nm and 645 nm, respectively. V is the volume of liquid to be measured (mL); m is leaf fresh mass (g) or leaf area (m^2^).

### 4.5. Measurement of Photosynthetic Parameters

Ci, Pn, Gs, and Tr were measured using LI-6400 a portable Photosynthesis Instrument 400 (LI-Cor6400XTPSC-4817, US). The first determination of leaves were to be numbered and labeled for the next determination.

### 4.6. Determination of Osmotic Regulator Content

Soluble sugar and soluble protein contents were determined using kits (Suzhou Keming Biotechnology Co., Ltd, Suzhou, China) according to the instructions.

Soluble sugar content determination: the absorbance value at A wavelength of 620 nm was measured after adding reagents to 0.1 g (W) leaves. The same volume of reagents was set as the control group for determination: soluble sugar (mg/g fresh weight) = 1.17 × (ΔA + 0.07) ÷ W, ΔA = A determination − A control.

Soluble protein content determination: the absorbance value at A wavelength of 620 nm was measured after adding reagent to 0.05 g (W) leaves. The same volume of reagent was set as the control group for determination: Cpr (mg/g fresh weight) = 0.1403 × (ΔA + 0.0007) ÷ W, ΔA = A determination − A control.

### 4.7. Data Analysis

SPSS 25 software was used for one-way analysis of variance and correlation analysis. Excel and Origin2021 software was used for data processing and mapping. PLS-DA analysis used https://www.metaboanalyst.ca/ (accessed on 12 March 2023.)

## Figures and Tables

**Table 1:** Correlation analysis of 13 indicators at 40 °C.

Indicator	H_2_O_2_	MDA	REC	SOD	POD	CAT	APX	Pn	Ci	Chla	Chlb	ss	sp	Gs	Tr
H_2_O_2_	1														
MDA	0.860 **	1													
REC	0.871 **	0.990 **	1												
SOD	0.017	0.425	0.426	1											
POD	−0.719 *	−0.463	−0.486	0.55	1										
CAT	0.975 **	0.940 **	0.936 **	0.187	−0.616	1									
APX	0.865 **	0.985 **	0.977 **	0.436	−0.411	0.940 **	1								
Pn	−0.752 *	−0.960 **	−0.961 **	−0.617	0.242	−0.854 **	−0.973 **	1							
Ci	0.226	0.142	0.116	−0.589	−0.680*	0.173	0.066	0.06	1						
Chla	−0.927 **	−0.971 **	−0.987 **	−0.327	0.57	−0.966 **	−0.957 **	0.916 **	−0.125	1					
Chlb	−0.957 **	−0.829 **	−0.848 **	−0.124	0.611	−0.932 **	−0.841 **	0.753 *	−0.009	0.914 **	1				
ss	−0.895 **	−0.946 **	−0.954 **	−0.39	0.427	−0.947 **	−0.979 **	0.941 **	−0.04	0.950 **	0.869 **	1			
sp	−0.930 **	−0.968 **	−0.969 **	−0.326	0.509	−0.979 **	−0.982 **	0.929 **	−0.122	0.971 **	0.884 **	0.988 **	1		
Gs	−0.590 *	−0.818 **	−0.837 **	−0.716 **	−0.065	−0.710 **	−0.888 **	0.933 **	0.326	0.791 **	0.564 *	0.872 **	0.832 **	1	
Tr	−0.615*	−0.855 **	−0.884 **	−0.745 **	−0.011	−0.734 **	−0.907 **	0.965 **	0.333	0.841 **	0.610 *	0.874 **	0.854 **	0.981 **	1

Note: ** and * mean significant difference at 0.01 and 0.05 levels, respectively.

**Table 2:** Effects of heat treatment at 40 °C for different days on the structure of peony leaves.

Days of Treatment (d)	LMA (g/m^2^)	LT/(μm)	LD (g/cm^2^)	PT/ST	PT/LT	ST/LT	MT/LT
0	95.00 ± 7.07ab	118.86 ± 9.53a	86.55 ± 11.91ab	0.70 ± 0.18b	0.32 ± 0.05bc	0.48 ± 0.07bc	2.84 ± 0.97b
1	92.53 ± 23.38ab	91.28 ± 10.75c	100.19 ± 15.11ab	0.59 ± 0.17bc	0.29 ± 0.05cd	0.5 ± 0.06ab	3.06 ± 0.29b
3	166.99 ± 86.38a	109.59 ± 12.06ab	137.03 ± 66.84a	0.75 ± 0.11b	0.34 ± 0.03b	0.45 ± 0.03c	5.31 ± 0.66a
5	106.94 ± 38.72ab	104.96 ± 7.63b	95.91 ± 33.59ab	1.03 ± 0.26a	0.38 ± 0.06a	0.38 ± 0.05d	3.06 ± 0.38b
7	62.50 ± 12.50b	90.03 ± 9.63c	64.62 ± 14.17b	0.50 ± 0.13c	0.26 ± 0.05d	0.53 ± 0.05a	2.19 ± 0.28b

Note: different letters in the same column indicate significant differences at the 0.05 level. Leaf mass per area (LMA, g·m^−2^); LMA= leaf dry weight/leaf area; leaf density (LD, g·cm^−3^); LD = LMA/LT.

## Data Availability

Not applicable.
